# Management of Erosion of Graft Materials in Pelvic Floor Reconstruction

**DOI:** 10.1100/tsw.2009.2

**Published:** 2009-01-18

**Authors:** Kathleen C. Kobashi

**Affiliations:** Continence Center at Virginia Mason, 1100 Ninth Avenue, Seattle, WA 98111, USA

**Keywords:** slings, erosion, extrusion, complications, mesh, polypropylene, prolapse repair

## Abstract

We present an overview of the current literature and management techniques for vaginal extrusion or urinary tract erosion of graft materials used in pelvic floor reconstruction. A MEDLINE search was performed to identify literature pertaining to the incidence and management of vaginal or urinary tract exposure of graft materials commonly used in anti-incontinence and pelvic floor reconstructive procedures. Dependent on the type of mesh material used, a vaginal extrusion rate of up to 77% has been reported. The currently accepted, loosely woven, monofilament type I polypropylene meshes appear to have acceptable lower exposure rates in the range of 1–3% for slings, but with the larger area of mesh used in prolapse repairs, the rate increases to up to 10%. With the current widespread use of graft materials to reinforce pelvic floor reconstructive techniques, it is imperative for surgeons to be familiar with potential complications related to the materials and proper management of these complications. Although it is beginning to appear that the benefit of using some synthetic materials may outweigh the risks, proper management and understanding of the risks is important in order to counsel our patients appropriately and responsibly prior to their surgeries.

## INTRODUCTION

The last decade has witnessed a remarkable movement toward the use of graft reinforcement for pelvic floor reconstruction. Historically, the first synthetic sling was reported by Williams and Telinde in 1962, who used mersiline mesh[1]; however, slings were almost exclusively constructed of biomaterials, primarily autologous tissues, until the 1990s when the introduction of several synthetic slings changed the field of pelvic floor reconstruction.

In an effort to improve on the efficacy and minimize the morbidity of traditional procedures, new approaches have emerged and techniques continue to evolve. A variety of synthetic materials have come into existence and the use of grafts has expanded from slings to prolapse repairs. With this introduction of synthetic meshes in pelvic floor surgery, new issues and sequelae have surfaced, among which are exposure of the graft into the vagina or the urinary tract, the management of which is the focus of this manuscript.

## MATERIALS AND METHODS

A comprehensive review of the literature using MEDLINE and sources cited in those peer-reviewed references was performed. Terms used in the search included “graft erosion”, “graft extrusion”, “pelvic floor reconstruction”, “anterior repair”, “posterior repair”, “colporrhaphy”, “urinary incontinence”, and “slings”. A review of the literature regarding erosion rates and management strategies is presented herein.

## RESULTS

Table 1 lists graft materials that have been used for pelvic floor reconstruction, their characteristics if known, and the reported respective range of erosions. As one can see, time at presentation of mesh extrusion is variable. Often this information is unknown, but clearly, patients may often not present immediately at onset of symptoms that might suggest extrusion. The literature contains reports of presentation of mesh extrusion ranging from 1 week to 40 months from the time of implantation[2,3]. Suffice it to say that mesh exposure can occur at any time postoperatively and studies with short-term follow-up may underestimate the rate of extrusion[4].

**TABLE 1**
**Synthetic Mesh Types, Characteristics, and Associated Vaginal Extrusion Rates***

| Material | Fiber Type | Pore Size | Extrusion Rate (%) | Ref. |
|---|---|---|---|---|
| Silicone | Monofilament | Macro | 40–71 | [14,15] |
| TVT™ (PP) | Monofilament | Macro | 2–3 | [2,18] |
| Prolapse repair (PP) | Monofilament | Macro | Up to 26 | [5] |
| ObTape™ (PP) | Monofilament | Micro | 5.4–16.6 | [10,11,12] |
| SPARC™/Monarc™ (PP) | Monofilament | Macro | 1.7–2.4 | [3,19] |
| Mersiline | — | — | 17 | [7] |
| IVS (PP) | Multifilament | Micro | 9 | [20] |

* Timing from implantation to presentation with extrusion varies from 1 week to 40 months[2,3].

In the case of type I polypropylene mesh (see Table 2), in spite of the use of similar materials, it appears that slings portend a lower risk of vaginal extrusion than graft-reinforced pelvic prolapse repair[5]. Intuitively, the risk of exposure would be proportional to the area of graft implanted and, hence, the higher rate of exposure noted with prolapse repair as compared to that seen with a simple sling. There is no indication that the pathophysiology of extrusion is different between slings and prolapsed repairs, nor does it appear that the location of the mesh (anterior or posterior) portends any difference in the risk of vaginal extrusion. Management of vaginal extrusion has ranged from observation to excision of the exposed portion of the graft to complete excision of the mesh.

## DISCUSSION

A variety of synthetic materials for slings and prolapse repair have been introduced over the years. Currently, the most widely accepted synthetic mesh used in pelvic floor reconstruction is a type I polypropylene mesh; however, data regarding morbidity and potential complications between various graft types, specifically with regard to anterior compartment repair, are still too scanty to provide reliable

**TABLE 2**
**Classification of Mesh Types**

| Type | Fiber | Pore Size |
|---|---|---|
| I | Monofilament | Macro (>75 μm) |
| II | Multifilament | Micro (<75 μm) |
| III | Multifilament | Variable |
| IV | Monofilament | "Submicro" |

comparison[6]. Table 2 illustrates the classification of mesh types. Although the incidence is low, these materials can erode into the urinary tract or extrude into the vagina. The rate at which these complications occur appears to be dependent on several factors, not the least of which is the type and weave of the material and factors such as porosity, flexibility, and coating of the material[7]. Management of graft erosion depends on the material involved and on the location and area of exposure. In general, the newer type I polypropylene meshes have provided an excellent graft option that seems to provide an acceptably low extrusion rate, a high success rate, and low morbidity that warrants their use.

Proposed risk factors for mesh extrusion or erosion include estrogen deficiency, improper dissection planes, poor tissue integrity, subclinical or frank infection, hematoma formation, increased mesh area, and lack of integration of the mesh into the host due to any of the above or related to a variety of mesh characteristics. Specifically regarding tissue dissection plane, undermining of the vaginal epithelium close to the surface can theoretically increase the risk of vaginal extrusion. Conversely, too deep a dissection can result in the more devastating urinary tract erosion. Nevertheless, it is the practice of the author to dissect a thicker epithelial layer than traditionally performed when using autologous tissue or other biograft.

Based on our experience with type I polypropylene mesh and that reported in the literature to date, it is the practice of the author to present the choice of observation with local hormone replacement therapy vs. formal excision of the exposed graft to the patient who presents with vaginal extrusion of mesh. It appears that spontaneous epithelialization over the graft may occur in some patients who choose observation[8]. Indeed, it has been proposed that indefinite observation without definitive intervention is safe in patients who are asymptomatic from vaginal mesh exposure of type I polypropylene mesh[9]. The same group described that patients who were sexually active were more likely to require mesh excision.

There are scant data on the effects of mesh removal on dyspareunia symptoms. It has not been reported that pain is exacerbated following excision, but the degree to which it resolves is also as yet undetermined. In patients in whom the mesh is being removed for pain, the question of how much mesh to remove arises. This, as well, is not universally established. There is no standard and, in actuality, there may never be, as each patient is unique on many levels. Although it may not be possible due to tissue in-growth, removal of the entire mesh in cases of pain, particularly when infection is suspected, may not relieve the pain. The reason for this is unclear, but inflammation and tissue changes related to the pelvic floor reconstruction and the presence of the mesh may not be reparable even with mesh removal.

In patients who are symptomatic from mesh exposure either themselves or via their sexual partners, and who do not wish to proceed with observation or in whom observation does not result in any decrease in the size of the defect within 6–8 weeks, definitive excision is recommended. Generally, in the specific case of type I polypropylene erosion, only the exposed portion of mesh need be excised. Although some groups have reported proceeding with an office procedure to excise the mesh, a formal visit to the operating suite is more likely to provide optimal exposure and a technically superior procedure.

At the edges of the exposure, the vaginal epithelium is often amenable to dissection off of the underlying mesh. The vagina is undermined and the plane behind the mesh is developed such that the mesh can be transected well away from (beneath) the vaginal edges. If the vagina is friable due to inflammation, tissue is excised until a healthy edge is accomplished. The undermining of the vaginal flaps is also important in facilitating a tension-free reapproximation of the vaginal tissue edges.

In cases of extrusion of non-type I mesh, complete removal of as much of the material as possible is recommended. Grafts that have shown themselves to be in this category include a thermally bonded polypropylene[10,11,12], a collagen-coated polyester[13], and silicone[14,15], among others. Often, a pseudocapsule or infection associated with these materials, which fail to be incorporated by the host tissue, allows the material to be removed without much dissection or difficulty. In fact, the mesh often simply slides out with gentle traction. Conversely, occasionally when this is not the case and the material seems to be fixed, every effort to dissect the graft free and remove it in its entirety should be exercised to avoid subsequent infection or eventual exposure of the retained fragment.

In the case in which any type of mesh is found in the bladder or urethra, complete excision of the mesh is warranted. These are cases in which the mesh has either been placed into the urinary tract or may have been placed under excess tension. Alternatively, any combination of dissection in a plane close to the bladder or urethra, excess tension of the graft, and poor tissue integrity due to estrogen deficiency, smoking, or other comorbidities could contribute to urinary tract graft exposure[16]. Removal of mesh can be exceedingly challenging due to tissue in-growth into the interstices of the graft. Often, the graft and involved tissue must be sharply removed[17]. The considerable area of bladder or urethral involvement may preclude recommendations to remove the entire mesh. Detailed discussion regarding urethral reconstructive techniques is beyond the scope of this manuscript, but suffice it to say that extensive mesh involvement in the urethra may require elaborate urethral reconstruction that would best be performed at a tertiary center. With bladder involvement, a transabdominal, transvesical approach is typically preferred with clean edges of bladder closed to itself. Careful bladder drainage should be accomplished via catheter for 7–10 days (typically both a urethral foley and a suprapubic tube), and catheter removal should be preceded by a cystogram with drainage film that confirms the absence of extravasation of contrast material.

## CONCLUSION

With the current widespread use of graft materials to reinforce pelvic floor reconstructive techniques, it is imperative for surgeons to be familiar with potential complications related to the materials and proper management of these complications. Although it is beginning to appear that the benefit of using some synthetic materials may outweigh the risks, proper management and understanding of the risks is important in order to counsel our patients appropriately and responsibly prior to their surgeries.
